# Cytoreductive surgery and Hyperthermic intra-operative peritoneal chemotherapy with Cisplatin for gastric peritoneal Carcinomatosis Monocentric phase-2 nonrandomized prospective clinical trial

**DOI:** 10.1186/s12885-017-3730-6

**Published:** 2017-11-17

**Authors:** Baki Topal, Karel Demey, Halit Topal, Joris Jaekers, Eric Van Cutsem, Vincent Vandecaveye, Xavier Sagaert, Hans Prenen

**Affiliations:** 1 Department of Abdominal Surgical Oncology, University Hospitals KU Leuven, Herestraat 49, 3000 Leuven, Belgium; 2 Department of Digestive Oncology, University Hospitals KU Leuven, Leuven, Belgium; 3 Department of Radiology, University Hospitals KU Leuven, Leuven, Belgium; 4 Department of Pathology, University Hospitals KU Leuven, Leuven, Belgium

## Abstract

**Background:**

**C**ytoreductive surgery (CRS) plus hyperthermic intra-operative peritoneal chemotherapy (HIPC) for gastric peritoneal carcinomatosis (PC) is controversial, and selection criteria for this treatment modality are lacking.

**Methods:**

Thirty-two patients (F/M ratio 12/20; median (range) age 58 (32-75) years) underwent CRS + HIPC with cisplatin for PC from gastric adenocarcinoma in 2010-2014. This monocentric phase-2 nonrandomized prospective study with a power of 90% aimed to improve the 1-year overall survival (OS) rate with 40% (historical reference of 52% to 72%). Median PCI score was 8 (range 1-20), number of regions involved was 6 (range 1-11). The impact of 16 prognostic factors on survival was evaluated using univariable and multivariable Cox regression models. Follow-up was complete in all patients, and closed 2 years after patient inclusion.

**Results:**

All patients had complete cytoreduction (CCR-0) and histopathological R0 resection. PCI </= 12 without PC on any small bowel region with 4 or more non-small bowel regions resulted in a median OS time of 24.7 months (15.6–29.4), and 1, 2, 5-year OS rates of 90%, 55%, 5.6%, respectively. Independent predictors of OS were PC on the small bowel combined with PC on 4 or more non-small bowel regions (*p* = 0.0004), number of regions involved (*p* = 0.0029), and overall PCI score (*p* = 0.0104).

**Conclusions:**

CRS + HIPC with cisplatin to treat gastric PC, providing complete cytoreduction and R0 resection, should be restricted to patients with PCI of 12 or less. Patients having PC on any small bowel region with 4 or more non-small bowel regions should be refused for CRS + HIPC.

**Trial registration number:**

Registration number: NCT01116791. Registration date: May 5, 2010.

## Background

Gastric cancer is a major health problem worldwide, with more than 950,000 new patients diagnosed every year and an estimated 720,000 deaths in 2012. Despite declining incidence and mortality for several decades, and despite substantial advances in understanding its epidemiology, pathogenesis, and therapeutic strategies, gastric cancer is still the fourth most common cancer and the second leading cause of cancer deaths worldwide [1].

Today, adequate surgical resection is the only chance for patients to be cured from gastric cancer, often in combination with peri-operative chemotherapy, or with postoperative chemo-radiotherapy [2–4]. However, only patients with localized disease are potential candidates for surgical management with curative intent. Most patients with gastric cancer present in an advanced stage with metastases in the peritoneal cavity and/or in the liver, and have an extremely poor prognosis. Peritoneal metastases, also called peritoneal carcinomatosis (PC), develop in up to 60% of gastric cancers and are a major cause of death [5]. Systemic combination chemotherapy is the standard of care for patients with metastasis, resulting in a median survival of about 8 months and almost no survivors at 3 years [6–8].

As the effect of systemic chemotherapy on gastric PC is unclear and is believed to be minimal, other treatment strategies have been studied aiming to improve survival of these patients. For about two decades, cytoreductive surgery (CRS) combined with hyperthermic intra-peritoneal chemotherapy (HIPC) has been used to treat PC from colorectal, ovarian, or mucinous appendiceal cancer, offering patients improved survival [9–11]. CRS + HIPC is now considered an important option in well-selected patients. Although CRS + HIPC has also been used in gastric PC, its beneficial effect on survival is still unclear and controversial. A recently published phase 3 randomized clinical trial on gastric PC showed improved survival after CRS + HIPC as compared with CRS alone, i.e. 11 vs 6.5 months (*p* = 0.046) [12]. The latest systematic reviews have demonstrated insufficient data to recommend CRS + HIPC as standard of care in gastric PC due to the lack of survival benefit and the heterogeneity among the published studies in terms of HIPC technique, duration, and cytotoxic agents used. Although a clear recommendation cannot yet be provided, evidence based on the literature suggests a potential role for CRS + HIPC in gastric PC [13, 14].

We conducted a monocentric phase-2 non-randomized clinical trial to evaluate long-term survival after CRS + HIPC with cisplatin in patients with gastric PC and to define selection criteria for this treatment modality.

## Methods

### Patients

Between August 2010 and November 2014, 41 patients with gastric PC signed informed consent forms to enter the study. At the time of surgery, 32 patients met inclusion criteria (Table 1), and underwent CRS + HIPC with cisplatin. At the time of surgical exploration, 9 patients were excluded from the study due to PCI >20. The male/female ratio was 20/12, with a median age of 58 years (range 32-75 y.). Patient comorbidity assessed by means of the American Society of Anaesthesiology (ASA) score was ASA II for 15 and ASA III for 17 patients. Patients’ median body mass index was 23.2 (range 15-34). Systemic cisplatin-based combination chemotherapy was administered in neo-adjuvant setting in 30 patients, of whom 21 were clinically fit to receive adjuvant chemotherapy within 3 months after surgery. Two patients (PCI score 6) refused neo-adjuvant systemic chemotherapy and were considered for primary surgery. One of these 2 patients was not fit for adjuvant chemotherapy due to postoperative anastomotic fistula and insufficient recovery from surgery.

**Table 1:** Selection criteria for CRS + HIPC for peritoneal carcinomatosis from gastric cancer

Inclusion criteria
− Primary or recurrent gastric adenocarcinoma
− Histological confirmation of peritoneal carcinomatosis from gastric adenocarcinoma
− Systemic chemotherapy and/or biological are allowed before and/or after CRS + HIPC
− Radiotherapy is allowed before or after CRS + HIPC
− Prior CRS + HIPC is allowed if performed more than 1 year ago
− Age between 18 to 75 years
− Patient Karnofsky Performance Scale (KPS) ≥ 80
− Signed informed consent
Exclusion criteria
− Pregnancy
− Any malignancy other than gastric adenocarcinoma
− Any metastatic disease other than peritoneal carcinomatosis, such as liver, pulmonary or bone metastases
− Peritoneal carcinomatosis index (PCI) > 20 at the start of CRS
− Impossibility to obtain complete cytoreduction (CCR-0) at the end of CRS
− Impossibility to obtain histopathological R0 resection at the end of CRS
− Clinical relevant ascites

CRS + HIPC was performed simultaneously with total gastrectomy in 25 patients with synchronous gastric PC, whereas 7 patients were treated for metachronous PC. Appendectomy and cholecystectomy were performed routinely in all patients. En-block distal pancreatectomy with splenectomy was performed in 5 patients because of macroscopic tumour invasion, and splenectomy alone in another 2 patients. No patient received radiotherapy, neither in neo-adjuvant nor in adjuvant setting.

Patient follow-up was complete in all patients, and ended in December 2016, 2 years after inclusion of the last patient in the study. Follow-up information was obtained through review of the patients’ hospital charts that were prospectively registered in our institution’s database. Postoperative follow-up investigations consisted of a clinical examination, biochemistry including serum carcinoembryonic antigen (CEA) level, abdominal ultrasound, contrast-enhanced computed tomography (CT), and/or magnetic resonance imaging (MRI) scan of the abdomen and thorax performed every 3–4 months.

#### Assessment of peritoneal carcinomatosis

Sugarbaker’s peritoneal cancer index (PCI) was used to assess peritoneal tumour burden of all 13 peritoneal regions [15]. Tumour burden and resectability prior to surgery were assessed using CT-scan of the abdomen and thorax, and whole-body diffusion MRI-scan with peritoneal protocol. Patients with clinically relevant ascites were excluded from the study, which was defined as ascites necessitating percutaneous trans-abdominal drainage or ascites throughout the entire peritoneal cavity measuring more than 1 cm width on CT-scan prior to CRS + HIPC. Diagnostic laparoscopy was employed routinely to evaluate PCI score and completeness of resectability before CRS + HIPC. At the time of laparotomy, two surgeons independently assessed resectability and scored the PCI to reach a consensus in case of different individual assessments. The overall median PCI score was 8 (range 1-20). The overall median number of regions involved in PC was 6 (range 1–11). PC of the small bowel was found in 24 patients, with a median score of 3 (range 1-8) and 2 (range 1-4) regions involved.

#### Surgical procedure

Cytoreductive surgery consisted of total gastrectomy with D2 lymphadenectomy (perigastric (D1) + celiac artery and its branches) in patients with synchronous gastric PC. The removal and histopathological analysis of at least 16 lymph nodes was aimed at to enable adequate tumour staging and to secure optimal surgical resection. Peritoneal carcinomatosis was treated by means of peritonectomy, electrofulguration of superficial (≤ 3 mm depth) metastases, and organ resection according to the surgeon’s judgment. Simultaneous colorectal resection was performed in 9 patients, splenectomy in 7, bilateral adnexectomy in 3, segmental small bowel resection in 5, distal pancreatectomy in 5, pancreaticoduodenectomy in 1, and bile duct resection followed by hepatico-jejunostomy in 1 patient.

An open coliseum technique was used for HIPC. Hyperthermic peritoneal chemotherapy was administered immediately after CRS, using cisplatin at a dose of 100 mg/m2 dissolved in 3-4 l of normal saline heated to 40° - 41° Celsius, and infused into the abdominal cavity for a sustained 60-min HIPC. Surgical reconstruction (anastomoses) was performed after HIPC. Immediately after surgery, patients were systematically monitored at the intensive care unit (ICU) for a median of 2 (range 0 – 12; IQR 1 - 3) days.

#### Outcome measures and prognostic factors

The primary endpoint was 1-year overall survival rate. Overall survival (OS) was defined as time from surgery to death, irrespective of cause. Disease-free survival (DFS) was defined as time to tumour recurrence or death, irrespective of cause. Peritoneal-DFS was defined as time to cancer recurrence at the peritoneal surface or death, irrespective of cause.

The impact of 16 potential prognostic factors on survival was evaluated: age, sex, ASA score, body mass index (BMI), synchronous or metachronous PC, preoperative systemic chemotherapy within 3 months before surgery, total PCI score, number of regions involved with PC, PC on the small bowel, small bowel PCI score, number of non-small bowel regions involved, non-small bowel PCI score, duration of surgery, amount of intra-operative blood loss, occurrence of postoperative complications, and postoperative systemic chemotherapy within 3 months after surgery.

In-hospital perioperative complications were studied as secondary endpoints. Postoperative complications were classified based on the therapy-oriented severity grading system (TOSGS) and allocated to surgical site (SSC) and non-surgical site complications (NSSC) [16].

#### Statistical analysis

Patients with gastric cancer suffering from PC and/or other metastases have OS rates ranging from 32% to 52% at 1 year [6–8]. The current study was conducted as a phase-2 monocentric prospective nonrandomized clinical trial and designed to have 90% power to detect 40% increase in 1y-OS rate after CRS + HIPC for PC from gastric cancer as compared to the previously reported historical reference of 52% 1y-OS rate [17]. Based on a simulation study, the number of study patients needed was calculated to be 27, and the target 1y-OS rate 72%. Minimal duration of follow-up after CRS + HIPC was fixed at 2 years. The anticipated period of patient inclusion was 3 years. Final survival analysis was planned at 2 years after inclusion of the last patient.

Kaplan-Meier estimates were used for survival analysis. Log-rank tests and Cox regression models were used to verify the relationship between a set of predictors and OS, DFS, and peritoneal-DFS, respectively. Median survival times until the event are reported with 95% confidence intervals (CI). A multivariable model was constructed combining the predictors with *p* < 0.10 in the univariable model for survival, irrespective of its significance. The proportional hazards assumption and the functional form of the continuous predictors were verified by applying graphical and numerical methods. *P*-values less than 0.05 were considered significant. All analyses were performed using JMP software, version 12.1.0 of the SAS Institute Inc. for Macintosh.

Follow-up was complete in all patients, and closed in December 2016, 2 years after the last patient was entered in the study.

#### Ethical considerations

The study was approved by the University Hospitals KU Leuven Ethical Committee prior to patient recruitment, and was given study number ML6615. The study was registered at clinicaltrials.gov under the number NCT01116791. This investigator-initiated study was conducted in accordance with the principles of the Declaration of Helsinki. Before enrolment into the study, written informed consent was obtained from all patients who fulfilled selection criteria.

## Results

### Postoperative outcome

Complete macroscopic cytoreduction (CCR-0) and histopathological R0 resection were obtained in all patients. Median duration of surgery was 300 (range 195 – 480; IQR 270 - 360) minutes, and intra-operative blood loss 300 (range 0 – 2600; IQR 100 – 500) millilitres.

No postoperative mortality occurred. Postoperative complications were observed in 23 (72%) patients, including 5 (16%) patients with transient cisplatin-associated nephrotoxicity without the need for haemodialysis (serum creatinine level > 2 mg/dL). According to the TOSGS score the severity of complications were grade 1 in 1, grade 2 in 13, grade 3a in 2, grade 3b in 4, grade 4a in 2, and grade 4b in 1 patient(s). Complications were allocated to SSC in 12, and NSSC in 16 patients. Median length of hospital stay (LOS) after surgery was 15 (range 9 – 70; IQR 13 - 26) days.

### Survival and prognostic factors

Median OS time after CRS + HIPC was 16.0 months (CI 12.2–24.5). The 1, 3, and 5-year OS rates were 71.9%, 14.1%, and 3.5%, respectively.

Median DFS and peritoneal-DFS times were 7.8 months (CI 6.4-10.7) and 10.7 months (7.1-12.8), respectively. The 1, 2, 3-year DFS and peritoneal-DFS rates were 25.8%, 6.4%, 6.4% and 41.4%, 14.0%, 14.0%, respectively.

**Fig. 1:**
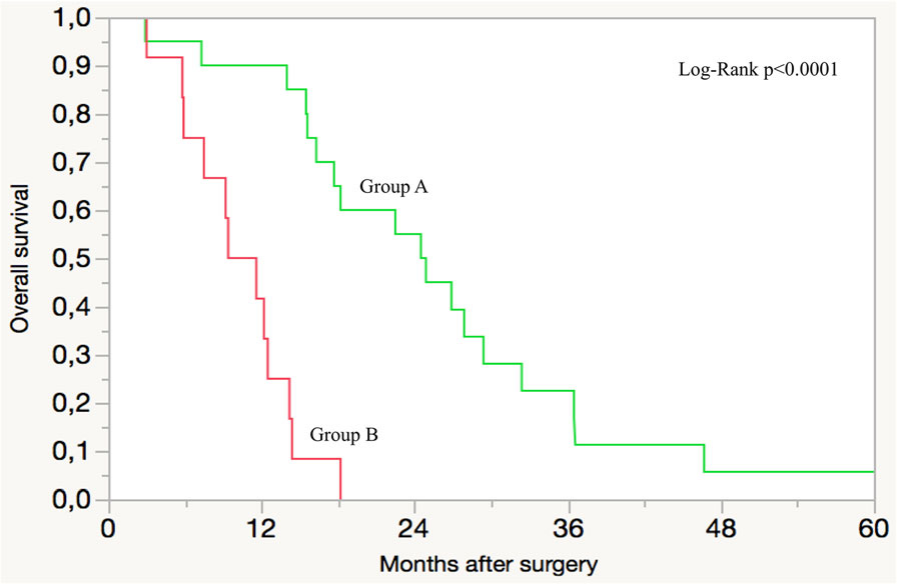
Overall survival rate after CRS + HIPC with cisplatin for peritoneal carcinomatosis from gastric cancer Group A: PCI </= 12 without PC on any small bowel region with 4 or more non-small bowel regions. Group B: PCI >/= 13 with PC on any small bowel with 4 or more non-small bowel regions. CRS + HIPC cytoreductive surgery plus hyperthermic intra-operative peritoneal chemotherapy; PC peritoneal carcinomatosis; PCI peritoneal carcinomatosis index

In univariable analyses several PC-related factors were significantly related to OS (Table 2). In multivariable analyses the presence of PC on the small bowel combined with PC on more than 3 non-small bowel regions (*p* = 0.0004), the overall number of regions involved with PC (*p* = 0.0029), and the total PCI score (*p* = 0.0104) were found as independent predictors of OS (Table 2). When variables were dichotomized, an overall PCI score of 13 or more, and the presence of PC on any small bowel region combined with PC on 4 or more non-small bowel regions were associated with worst OS (Fig. 1). Patients having these variables had a median OS time of 10.5 months (5.8 – 10.2), a 1-year OS rate of 41.7%, and they all died within 18 months after CRS + HIPC. Patients having a total PCI score of 12 or less, without having PC on any small bowel region with 4 or more non-small bowel regions had the best survival. Their median OS time was 24.7 months (15.6 – 29.4), and 1,2, and 5-year OS rates 90%, 55%, and 5.6%, respectively (*p* < 0.0001). They also had a significantly better median DFS (9.7 vs. 6.6 months; *p* = 0.034) and a peritoneal-DFS (12.6 vs. 6.9 months; *p* = 0.002) as compared to other patients.

**Table 2:** Results of univariable and multivariable Cox regression models for overall survival

		N	Median OS	Univariable		Multivariable	
				Hazard ratio	*p* value	Hazard ratio	*p* value
Age (years)					0.260		
Gender					0.712		
BMI					0.122		
ASA	2	15	12.2 (5.8-14.4)		*0.037*	0.170 (-0.299-0.638)	0.4730
	3	17	18.2 (15.5-27.9)				
Synchronous PC					0.999		
Neo-adjuvant chemotherapy	No	2	8.6 (3-14.2)		0.054	0.443 (-0.562-1.281)	0.3459
	Yes	30	17 (12.5-24.9)				
Duration of surgery (minutes)					0.444		
Intra-operative blood loss (ml)					0.619		
Total PCI score				0.114 (0.018-0.212)	*0.020*	0.750 (0.180-1.344)	*0.0104*
Number of regions involved PC				0.167 (0.005-0.340)	*0.042*	1.290 (0.434-2.246)	*0.0029*
Small bowel PCI score					0.1511		
Non-small bowel PCI score					0.068	0.564 (0.088-1.226)	0.0888
PC on small bowel	No	8	28.6 (14-36.6)		0.098	0.545 (0.195-1.268)	0.1453
	Yes	24	14.3 (9.2-18.2)				
Number of small bowel regions involved PC					0.497		
Number of non-small bowel regions involved PC					0.078	0.910 (0.118-2.008)	0.0831
PC on any small bowel region with 4 or more non-small bowel regions	No	20	24.7 (15.6-29.4)		*<0.0001*	1.383 (0.624-2.177)	*0.0004*
	Yes	12	10.5 (5.8-14.2)				
Adjuvant chemotherapy					0.821		
Postoperative complications					0.502		

95% confidence intervals are mention between parentheses; OS times are mentioned in months

*ASA* American society of anesthesiology, *BMI* body mass index, *ml* milliliters, *N* number, *OS* overall survival, *PC* peritoneal carcinomatosis, *PCI* peritoneal carcinomatosis index

Independent predictors of OS were PC on the small bowel combined with PC on 4 or more non-small bowel regions (*p* = 0.0004), number of regions involved (*p* = 0.0029), and overall PCI score (*p* = 0.0104)

Peritoneal cancer recurrence was observed in 24 (75%) patients, whereas 30 (94%) patients developed cancer recurrence at any location (liver, peritoneal, skeletal, distant lymph node metastases). Although in univariable analyses PCI scores and numbers of regions involved were significantly related to DFS and peritoneal-DFS, in multivariable analyses none of these variables were found to be independent predictors of either DFS or peritoneal-DFS (*p* > 0.15).

## Discussion

Our study demonstrates that CRS + HIPC with cisplatin can improve survival of selected patients with PC from gastric cancer, with acceptable morbidity and no mortality. We found 3 variables that determine survival and define the best candidates for CRS + HIPC. In patients having PCI scores of 12 or less without PC on any small bowel region with 4 or more non-small bowel regions, CRS + HIPC resulted in a median OS time of 24.7 months and a 1-year OS rate of 90%. These patients also had significantly better DFS and peritoneal-DFS outcomes as compared to other patients treated with CRS + HIPC. Patients having PCI scores of 13 or more and the presence of PC on any small bowel region with 4 or more non-small bowel regions died within 18 months after CRS + HIPC. In the only phase 3 randomized clinical trial published so far, the median OS time after CRS + HIPC for gastric PC was 10.2 months in the low PCI group (PCI < 20; *n* = 20), and 13.5 months in the high PCI group (PCI > 20; *n* = 14). The authors also found the completeness of cytoreduction to determine survival. CRS + HIPC plus CCR-0-1 (n = 20) was associated with median OS time of 12.0 months [12]. These figures seem to be inferior to the median OS of 16 months in our study, which might be explained by tumour biology, the fact we obtained a CCR-0 status in all our patients, and on histopathological examination an R0 resection was obtained in all patients. The peri-operative mortality rate in our patient population was zero, just like it was in the study of Yang et al. [12].

Our survival data seem to be better than those reported with any other treatment modality for PC from gastric cancer, and may have several explanations. A recent systematic review that focused on survival, mortality, and morbidity of CRS + HIPC for PC from gastric cancer showed significant heterogeneity in the studies, which might bias final conclusions (441 patients from 3 retrospective and 7 prospective studies published between 2000 and 2010) [13]. Although contrasting data were presented about survival rates, all findings pointed to the necessity of complete cytoreduction (CCR-0) to improve survival. With a relatively high postoperative mortality rate of about 5%, the published median 1-y OS rate was 43% (range 22 – 68%). Given these results, major controversy had arisen about the role of CRS + HIPC, as current systemic chemotherapy without surgery offers patients with metastatic gastric cancer 1-y OS rates of up to 52% [17].

Peritoneal carcinomatosis is present in about one third of patients presenting with metastatic gastric cancer, and is associated with a median survival time of about 8 months, 1-year survival rate of around 50%, and almost no survivors at 3 year despite systemic chemotherapy [7, 8]. Indeed, to date, systemic chemotherapy is the standard of care for patients suffering from gastric PC. In some cases, peritoneal carcinomatosis may be the only site of dissemination, and CRS + HIPC may be a therapeutic option. In colon or appendiceal cancer with isolated PC, a benefit is suggested for CRS + HIPC, whereas its role in gastric cancer is less clear. In the absence of randomized controlled trials, several cohort studies addressed the use of CRS + HIPC for peritoneal carcinomatosis from gastric cancer. In our study, the 1-y OS rate was 90% for patients having favourable prognostic factors, and seems better than the reference of 52%. Completeness of cytoreduction (CCR-0) and R0-resection may contribute to this favourable outcome. Other factors with a positive effect on survival may be the absence of postoperative mortality, appropriate selection of patients, and high rates of systemic chemotherapy in neo-adjuvant and adjuvant settings. As our study is a phase-2 nonrandomized prospective clinical trial, its promising survival warrants further study in future phase-3 randomized controlled trials to investigate whether the survival advantage is due to tumour biology or to CRS + HIPC. Furthermore, it should be taken into account that quality of surgery and its clinical outcome depend on the skills of the surgical team and might therefore not be replicated in routine clinical practice, but would need to be limited to experienced centres. The extent of PC appears to have a negative effect on survival. We found PCI scores greater than 12 and the presence of PC on small bowel combined with PC on more than 3 non-small bowel regions as criteria not eligible for CRS + HIPC. Studies on PC from other cancer types in some series support a good outcome for patients with a maximum PCI of 16 [18].

The potential role of CRS + HIPC in patients without overt peritoneal metastases is also under debate. Although patients with localized gastric cancer are treated with curative intent by means of radical surgery with either neo-adjuvant or adjuvant systemic chemotherapy, many of them will finally develop PC resulting in fatal outcome. In the absence of macroscopic peritoneal metastases, staging laparoscopy with peritoneal lavage showing positive cytology results in poor survival and is defined as metastatic disease [19]. Therefore, the addition of CRS + HIPC in the therapeutic armamentarium of surgical oncologists to treat advanced gastric cancer without clinically overt metastases is worth studying in future randomized controlled trials.

The morbidity rate and the severity of complications in our study were in accordance with those of other studies, but there was no mortality. Despite adequate hydration in the perioperative stage, transient cisplatin-associated nephrotoxicity was observed in 16% of patients. At the time our study was started, cisplatin was the standard of care as systemic chemotherapy both in neo-adjuvant as in adjuvant setting in potentially curable patients with operable gastric cancer, and in palliative setting for metastatic disease. Today, the administration of oxaliplatin in HIPC, as often used in PC from colorectal cancer, might be an alternative since systemic oxaliplatin is less nephrotoxic and as effective as systemic cisplatin in advanced esophagogastric cancer [20]. However, whether oxaliplatin could provide similar survival outcomes as cisplatin used in CRS + HIPC for gastric PC, needs to be evaluated in future studies.

## Conclusion

In patients with limited PC from gastric cancer, CRS + HIPC with cisplatin results in favourable survival, provided a complete cytoreduction (CCR-0) and histopathological R0 resection are obtained. It should be restricted to patients having PCI scores of 12 or less. Patients having PC on any small bowel region with PC on 4 or more non-small bowel regions should be refused for CRS + HIPC.

## Abbreviations

CCR: Completeness of cytoreduction
CRS: Cytoreductive surgery
DFS: Disease-free survival
HIPC: Hyperthermic intra-operative peritoneal chemotherapy
OS: Overall survival
PC: Peritoneal carcinomatosis
PCI: Peritoneal carcinomatosis index

## References

[1] Stewart BW, Wild CP (2014). World cancer report.

[2] Macdonald JS, Smalley SR, Benedetti J, Hundahl SA, Estes NC, Stemmermann GN, Haller DG, Ajani JA, Gunderson LL, Jessup JM, Martenson JA (2001). Chemoradiotherapy after surgery compared with surgery alone for adenocarcinoma of the stomach or gastroesophageal junction. N Engl J Med.

[3] Cunningham D, Allum WH, Stenning SP, Thompson JN, Van de Velde CJ, Nicolson M, Scarffe JH, Lofts FJ, Falk SJ, Iveson TJ, Smith DB, Langley RE, Verma M, Weeden S, Chua YJ, MAGIC Trial Participants (2006). Perioperative chemotherapy versus surgery alone for Resectable Gastroesophageal cancer. N Engl J Med.

[4] Van Cutsem E, Sagaert X, Topal B, Haustermans K, Prenen H (2016). Gastric cancer. Lancet.

[5] Nakamura K, Ueyama T, Yao T, Xuan ZX, Ambe K, Adachi Y, Yakeishi Y, Matsukuma A, Enjoji M (1992). Pathology and prognosis of gastric carcinoma. Findings in 10,000 patients who underwent primary gastrectomy. Cancer.

[6] Wagner AD, Unverzagt S, Grothe W, Kleber G, Grothey A, Haerting J, Fleig WE (2010). Chemotherapy for advanced gastric cancer. Cochrane Database Syst Rev.

[7] Ajani J, Rodriguez W, Bodoky G, Moiseyenko V, Lichinitser M, Gorbunova V, Vynnychenko I, Garin A, Lang I, Falcon S (2010). Multicenter phase III comparison of cisplatin/S-1 with cisplatin/infusional fluorouracil in advanced gastric or gastroesophageal adenocarcinoma study: the FLAGS trial. J Clin Oncol.

[8] Thomassen I, van Gestel YR, van Ramshorst B, Luyer MD, Bosscha K, Nienhuijs SW, Lemmens VE, de Hingh IH (2014). Peritoneal carcinomatosis of gastric origin: a population-based study on incidence, survival and risk factors. Int J Cancer.

[9] Vanounou T, Garfinkle R (2016). Evaluation of Cytoreductive surgery and Hyperthermic Intraperitoneal chemotherapy for peritoneal Carcinomatosis of colorectal origin in the era of value-based medicine. Ann Surg Oncol.

[10] Huo YR, Richards A, Liauw W, Morris DL (2015). Hyperthermic intraperitoneal chemotherapy (HIPEC) and cytoreductive surgery (CRS) in ovarian cancer: a systematic review and meta-analysis. Eur J Surg Oncol.

[11] KJ K, Nash GM (2014). Peritoneal debulking/intraperitoneal chemotherapy-non-sarcoma. J Surg Oncol.

[12] Yang XJ, Huang CQ, Suo T, Mei LJ, Yang GL, Cheng FL, Zhou YF, Xiong B, Yonemura Y, Li Y (2011). Cytoreductive surgery and Hyperthermic Intraperitoneal chemotherapy improves survival of patients with peritoneal Carcinomatosis from gastric cancer: final results of a phase III randomized clinical trial. Ann Surg Oncol.

[13] Gill RS, Al-Adra DP, Nagendran J, Campbell S, Shi X, Haase E, Schiller D (2011). Treatment of gastric cancer with peritoneal carcinomatosis by cytoreductive surgery and HIPEC: a systematic review of survival, mortality, and morbidity. J Surg Oncol.

[14] Montori G, Coccolini F, Ceresoli M, Catena F, Colaianni N, Poletti E, and Ansaloni L. The treatment of peritoneal carcinomatosis in advanced gastric cancer: state of the art. Int J Surg Oncol 2014, 912418. doi:10.1155/2014/912418. 10.1155/2014/912418 PMC3947693 24693422

[15] Sugarbaker PH (1995). Prognostic features of 51 colorectal and 130 appendiceal cancer patients with peritoneal carcinomatosis treated by cytoreductive surgery and intraperitoneal chemotherapy. Ann Surg.

[16] Dindo D, Demartines N, Clavien PA (2004). Classification of surgical complications: a new proposal with evaluation in a cohort of 6336 patients and results of a survey. Ann Surg.

[17] Boku N, Yamamoto S, Fukuda H, Shirao K, Doi T, Sawaki A, Koizumi W, Saito H, Yamaguchi K, Takiuchi H, Nasu J, Ohtsu A, Gastrointestinal Oncology Study Group of the Japan Clinical Oncology Group (2009). Fluorouracil versus combination of irinotecan plus cisplatin versus S-1 in metastatic gastric cancer: a randomised phase 3 study. Lancet Oncol.

[18] Vaira M, Cioppa T, D’Amico S, de Marco G, D'Alessandro M, Fiorentini G, De Simone M (2010). Treatment of peritoneal carcinomatosis from colonic cancer by cytoreduction, peritonectomy and hyperthermic intraperitoneal chemotherapy (HIPEC). Experience of ten years. In Vivo.

[19] Mezhir JJ, Shah MA, Jacks LM, Brennan MF, Coit DG, Strong VE (2010). Positive peritoneal cytology in patients with gastric cancer: natural history and outcome of 291 patients. Ann Surg Oncol.

[20] Cunningham D, Starling N, Rao S, Iveson T, Iveson T, Nicolson M, Coxon F, Middleton G, Daniel F, Oates J, Norman AR, Upper Gastrointestinal Clinical Studies Group of the National Cancer Research Institute of the United Kingdom (2008). Capecitabine and oxaliplatin for advanced esophagogastric cancer. N Engl J Med.

